# Potential Anti-Inflammatory and Anti-Cancer Properties of Farnesol

**DOI:** 10.3390/molecules23112827

**Published:** 2018-10-31

**Authors:** Young Yun Jung, Sun Tae Hwang, Gautam Sethi, Lu Fan, Frank Arfuso, Kwang Seok Ahn

**Affiliations:** 1 Department of Science in Korean Medicine, Kyung Hee University, 24 Kyungheedae-ro, Dongdaemun-gu, Seoul 02447, Korea; ve449@naver.com (Y.Y.J.); suntaeh12@gmail.com (S.T.H.); 2 Department of Pharmacology, Yong Loo Lin School of Medicine, National University of Singapore, Singapore 117600, Singapore; phcfanl@nus.edu.sg; 3 Stem Cell and Cancer Biology Laboratory, School of Pharmacy and Biomedical Sciences, Curtin Health Innovation Research Institute, Curtin University, Perth WA 6009, Australia; frank.arfuso@curtin.edu.au; 4 Department of Korean Pathology, College of Korean Medicine, Kyung Hee University, 24 Kyungheedae-ro, Dongdaemun-gu, Seoul 02447, Korea

**Keywords:** farnesol, inflammation, cancer, apoptosis

## Abstract

Farnesol, an acyclic sesquiterpene alcohol, is predominantly found in essential oils of various plants in nature. It has been reported to exhibit anti-cancer and anti-inflammatory effects, and also alleviate allergic asthma, gliosis, and edema. In numerous tumor cell lines, farnesol can modulate various tumorigenic proteins and/or modulates diverse signal transduction cascades. It can also induce apoptosis and downregulate cell proliferation, angiogenesis, and cell survival. To exert its anti-inflammatory/anti-oncogenic effects, farnesol can modulate Ras protein and nuclear factor kappa-light-chain-enhancer of activated B cells activation to downregulate the expression of various inflammatory mediators such as cyclooxygenase-2, inducible nitric oxide synthase, tumor necrosis factor alpha, and interleukin-6. In this review, we describe the potential mechanisms of action underlying the therapeutic effects of farnesol against cancers and inflammatory disorders. Furthermore, these findings support the clinical development of farnesol as a potential pharmacological agent in clinical studies.

## 1. Introduction

**Figure 1:**
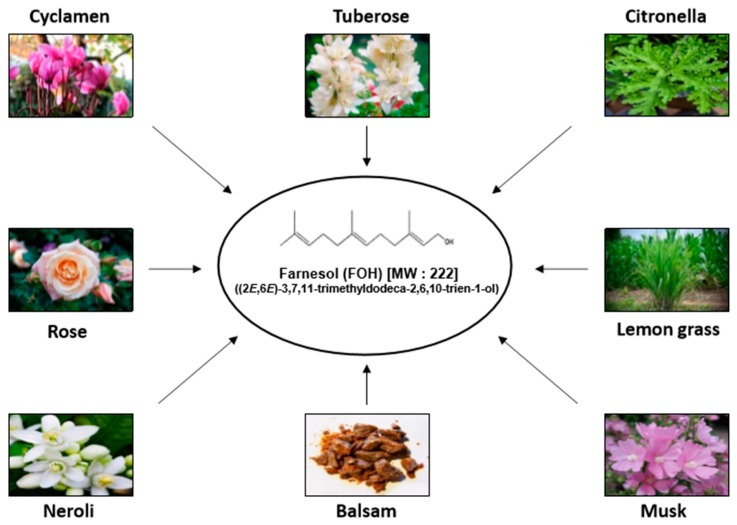
Various natural sources of farnesol.

**Figure 2:**
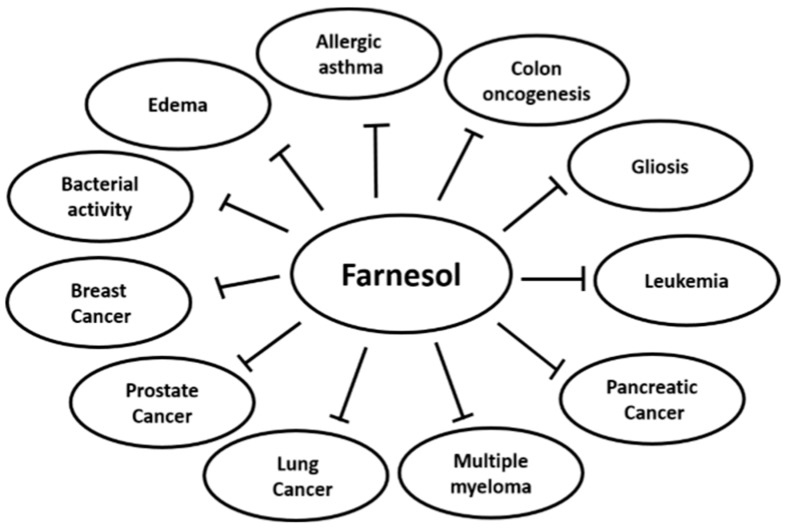
Effects of farnesol on cancer and inflammatory disorders.

In contemporary disease, synthetic drugs play essential roles in both prevention and cure; however, several of these may affect normal homeostasis and exhibit substantial adverse effects. On the other hand, various compounds extracted from natural sources, such as fruits, vegetables, and plants, have been used for chemoprevention because they may have relatively lower toxicity as compared to their synthetic counterparts [1,2,3,4]. Farnesol is a constituent of essential oil derived from various plants such as citronella, lemon grass, tuberose, cyclamen, rose, neroli, balsam, and musk (Table 1) (Figure 1) [5,6,7,8,9]. It is a non-sterol isoprenoid (sesquiterpenoid alcohol) that can also be produced endogenously by the dephosphorylation of farnesyl pyrophosphate in the cholesterol biosynthesis pathway [10]. Farnesol has a chemical formula of C_15_H_25_OH and a molecular mass of 222.372 g/mol. Exogenous farnesol has been used to treat allergic asthma, diabetes, atherosclerosis, obesity, and hyperlipidemia [11,12,13,14]. Farnesol has been reported to regulate inflammatory responses and has a beneficial effect with edema, allergic asthma, gliosis, skin tumorigenesis, colon oncogenesis, and the immune response system (Figure 2) [14,15,16,17,18,19,20,21]. Also, farnesol has shown anti-neoplastic effects in various human cancers, such as prostate cancer, breast cancer, lung cancer, pancreatic cancer, and multiple myeloma, by inhibiting cell proliferation in vitro and suppressing tumor growth in vivo (Figure 2) [22,23,24,25,26,27,28,29,30,31,32,33,34,35]. Moreover, farnesol containing cream has been reported to exhibit beneficial effect in atopic dermatitis patients [36], but farnesol preparations can also cause allergic reactions as contact allergen when used as fragrance material, as reported in clinical studies [37,38,39]. Thus, the toxicity of pharmacological preparations containing farnesol has to be carefully monitored.

One of the processes that predominantly drive tumor progression is inflammation. Although acute inflammation is part of the earliest responses to tissue damage, and it helps to counter against the invasion of foreign pathogens, chronic inflammation can create an environment that supports tumor initiation and development [40,41]. Chronic inflammation in the tumor microenvironment also allows for tumor invasion and metastasis to occur as pro-inflammatory mediators can provide signal to tumor cells to extravasate into the stroma [41]. Additionally, chronic inflammation can also drive major diseases such as atherosclerosis, asthma, and Alzheimer’s disease [41]. The mechanism(s) by which chronic inflammation promotes these diseases, especially cancer, is through the continuous production of cytokines and chemokines, such as tumor necrosis factor alpha (TNFα), interleukin (IL)-1, IL-6, and C–X–C chemokine receptor type 4, through the constitutive activation of nuclear factor kappa-light-chain-enhancer of activated B cells (NF-κB), which is a pro-inflammatory transcription factor [40,41]. The inhibition of the pro-inflammatory NF-κB signaling pathway in various cancers has proven to effectively inhibit tumor growth, invasion, and metastasis; for example in hepatocellular carcinoma, colorectal, breast, and prostate cancer [42]. However, a total inhibition of inflammation can also worsen cancer prognosis, as noted from the use of TNFα blockers resulting in an increased risk of lymphoma development [40]. Therefore, regulation of inflammation in anti-cancer treatment should strive to ameliorate chronic inflammation to maintain the beneficial acute inflammatory state. Since farnesol has been shown to be able to regulate inflammation in several diseases and also inhibit tumor growth in various cancers, the inflammation-modulatory and anti-cancer effects of farnesol that have been reported in prior studies have been briefly summarized in this review.

## 2. In Vitro Inflammatory-Modulatory and Anti-Tumor Effects of Farnesol

### 2.1. In Vitro Pro-Inflammatory Effects of Farnesol

**Figure 3:**
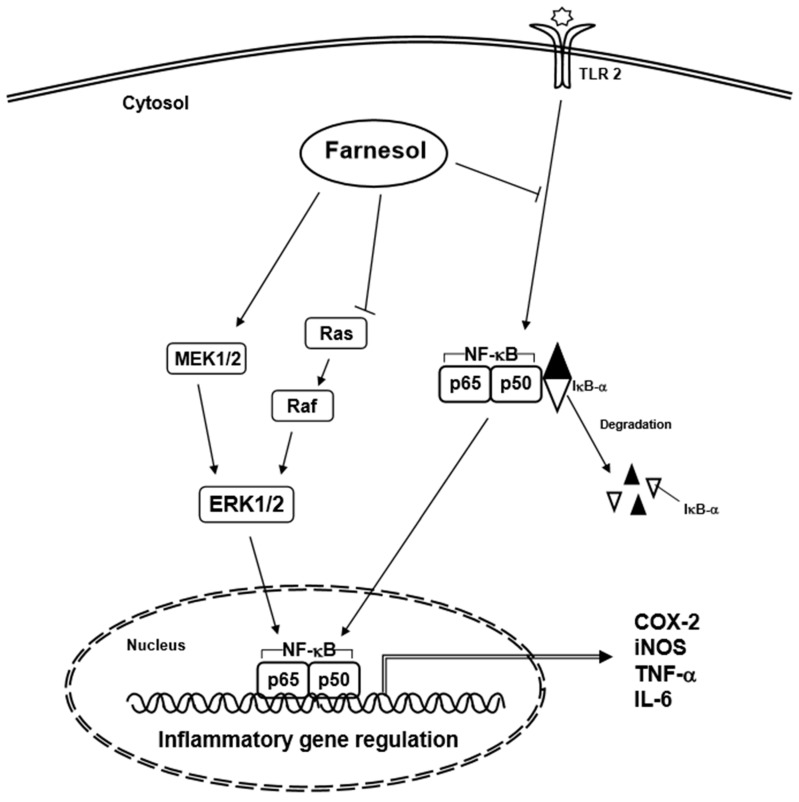
Inflammatory signaling pathways affected by farnesol.

Farnesol has been found to stimulate the immune system response through upregulating the expression of pro-inflammatory genes such as *IL-6*, *TNF-α*, and *cyclooxygenase-2* (*COX-2*) in human lung adenocarcinoma cells and murine macrophage cells (Figure 3) [15,16]. These genes are controlled by NF-κB, which can also regulate the expression of a variety of genes involved in the immune-inflammatory response, including those related to pro-inflammatory activity [43]. NF-κB expression is regulated by nuclear factor of kappa light polypeptide gene enhancer in B cells inhibitor, alpha (IκB-α) [43]. Farnesol can reduce the level of IκB-α protein, leading to an increase of the immune response in human lung adenocarcinoma cells (H460) (Table 2) [16]. In addition, farnesol can induce the activation of mitogen-activated protein kinase (MAPK) signaling pathways, especially activation of MEK1/2-ERK1/2 [16]. Generally, activation of the MEK-ERK signaling pathway is a pro-survival signal, but activation of the MEK1/2-ERK1/2 pathway can also result in the induction of apoptosis [16]. Farnesol can induce phosphorylation of p65, which is dependent on activation of the MEK1/2-ERK1/2 signaling pathway in human lung adenocarcinoma cells (H460) [16], leading to increased p65 activity and chemokine ligand 3, IL-1, and COX-2 expression [16]. In addition, farnesol has shown to exert its pro-inflammatory effect by increasing the levels of IL-6, TNF-α, and COX-2 in macrophage cells (RAW 264.7) (Table 2) [15]; these effects were markedly augmented when farnesol was combined with zymosan [15]. In an in vitro study carried out with primary murine splenocytes, farnesol treatment did not have an anti-inflammatory effect on the cells as it did not cause a significant change in the ratio of Th2/Th1 cytokines, as well as IL-10 and IL-2 production respectively, as compared to other sesquiterpenoid compounds [44].

### 2.2. In Vitro Anti-Inflammatory Effects of Farnesol

Apart from being produced by various plants, farnesol has also been excreted as a quorum sensing molecule by the fungus *Candida albicans* [45]. It also acts as a virulence factor, with mutants producing less and more farnesol becoming less and more infectious respectively [45]. The defense mechanism of the host organism in response to *C. albicans* infection can be usually mediated through the activation of acute inflammation, with Th1 cells producing pro-inflammatory cytokines to clear the infection [45]. Farnesol can function as a virulence factor by causing an anti-inflammatory response and suppressing pro-inflammatory cytokines, which makes the host organism more susceptible to the infection [45]. An investigation carried out with primary murine macrophages suggested that farnesol can also reduce the production of IL-12, which is a cytokine necessary for the differentiation of naïve T cells to Th1 cells as well as to stimulate the production of the pro-inflammatory factor, interferon gamma [45]. Therefore, farnesol may suppress immunity against *C. albicans* infection through the modulation of the inflammatory response.

### 2.3. In Vitro Anti-Tumor Effects of Farnesol

**Figure 4:**
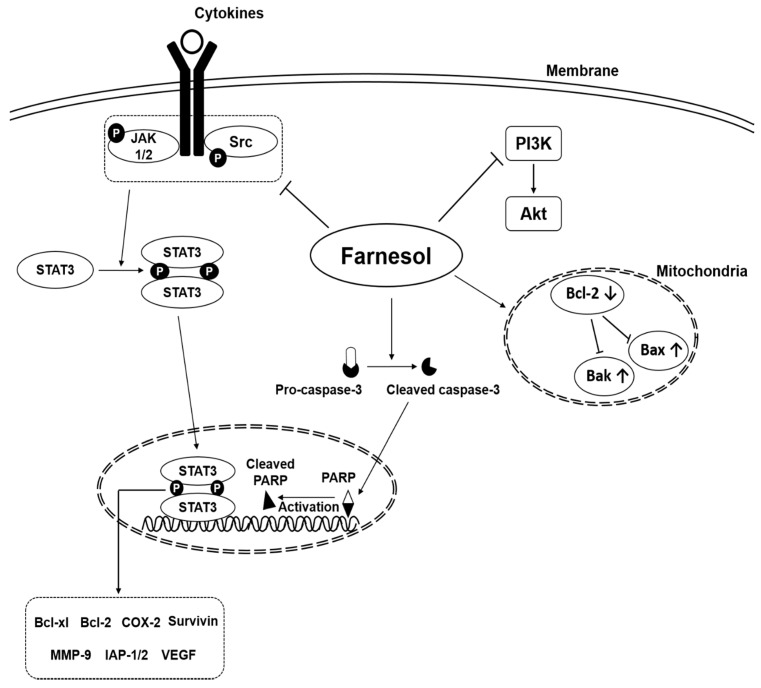
Oncogenic signaling pathways modulated by farnesol.

Natural products have attracted significant attention for their anti-tumor effects since several years [12,22,23,24,25,26,27,28,29,30,31,32,33,34,35,46,47,48,49,50,51,52,53,54,55,56,57]. Farnesol is one compound that has been reported to downregulate cell proliferation and angiogenesis, and to induce apoptosis through targeting various molecular targets in several tumor cell lines such as prostate, breast, lung, pancreas, cervical, oral squamous cell, meningioma, multiple myeloma, and T lymphoblastic leukemia (Table 2) (Figure 4) [22,23,24,25,26,27,28,29,30,31,32,33,34,35].

### 2.4. Prostate Cancer

In diverse tumor cell lines, the phosphatidylinositol-3-kinase (PI3K) and serine/threonine kinase (Akt) signaling pathway is essential to regulate cell proliferation, cell survival, and apoptosis [46,47]. Activation of the PI3K and Akt signaling pathway inhibits the efficacy of chemotherapeutic drugs in various tumor cell lines [48]. Many reports have shown that inhibition of the PI3K and Akt signaling pathway facilitates chemotherapy through the induction of apoptosis in prostate cancer cells [22,49,50,51]. The MAPK family consists of three major members such as p38, ERK, and JNK, which respond to growth factors, cytokines, and stress to interfere with intracellular signaling associated with cell proliferation, cell death, cell survival, and transformation [52,53]. Farnesol-induced apoptosis in prostate DU145 cells was examined by Annexin V/propidium iodide staining [22]. Following treatment with farnesol, the protein levels of activated p-JNK, p-ERK, p-p38, p-Akt, and apoptosis-related signals including p53, Bcl-2, Bax, and cleaved caspase-3 were decreased [22]. Additionally, when LNCaP and PC-3 prostate cancer cells were treated with a farnesol and ibandronate combination, cell growth was inhibited; furthermore, farnesol alone appeared to be a potent inhibitor of tumor cell growth [23].

### 2.5. Breast Cancer

Duncan et al. reported that farnesol can induce the expression of thyroid hormone receptor (THR) β1, which inhibited cell growth in breast cancer cell lines [24]. Also, farnesol activated nuclear hormone receptors, such as farnesoid X receptor and peroxisome proliferator activated receptor-α/γ (PPARα, and PPARγ), which are steroid/thyroid nuclear receptor superfamily members that can regulate gene transcription [12,54,55]. In MCF-7 breast cancer cells, farnesol inhibited cell growth and induced THRβ1 protein/mRNA levels in a concentration- and time-dependent manner, but this effect was not observed in MDA-MB-231 breast cancer cells [24].

### 2.6. Lung Cancer

Using an XTT assay, it was found that farnesol reduced the cell viability of A549 and H460 lung cancer cells [25,27]. In the A549 cell line, farnesol treatment caused a cell cycle arrest of the cells in the G0/G1 phase, which subsequently resulted in apoptosis of the cells [26]. Since farnesol is structurally similar to the substrate of protein prenylation, farnesyl pyrophosphate, it was hypothesized that farnesol could act as a competitive inhibitor of prenyltransferases and inhibit the prenylation of Ha-Ras, a small G-protein, to suppress the activation of the Ras/Raf/ERK pathway [26]. However, it was demonstrated that farnesol-induced cell cycle arrest and apoptosis of the A549 cells were independent of the ERK pathway and farnesol did not affect the prenylation of Ha-Ras [26]. Alternatively, the anti-tumor effects of farnesol on the A549 cell line were due to its inhibition of the phosphatidylcholine (PC) biosynthesis pathway, where it suppressed the conversion of cytidine diphosphate-choline to PC, a step regulated by choline phosphate transferase (CPT) [26]. The substrate of CPT is diacylglycerol (DAG), and farnesol was also able to inhibit the activity of CPT by competitive inhibition with DAG. Addition of DAG rescued CPT inactivation by farnesol as well as preventing cell cycle arrest and apoptosis of the A549 cells [26]. In a previous study, farnesol was shown to induce the disorganization of the actin cytoskeleton in A549 cells, but addition of exogenous DAG and PC blocked its disorganization [26,58]. Moreover, supplementing the cells with exogenous PC was also able to attenuate the G0/G1 phase cell cycle arrest and apoptosis of farnesol-treated A549 cells, thus demonstrating that farnesol may also exert its anti-tumor effects through the regulation of PC biosynthesis [26].

In another lung cancer cell line, H460, farnesol treatment induced endoplasmic reticulum (ER) stress, which triggered the unfolded protein response (UPR) and caused apoptosis of the cells through the intrinsic apoptotic pathway, where there was an increase in the cleavage of caspase-9, caspase-3, and poly (ADP-ribose) polymerase (PARP), but not caspase-8 [27]. Farnesol treatment initiated the activation of the UPR signaling cascade, namely the PKR-like ER kinase (PERK)-eIF2α and the inositol requiring protein 1 (IRE1)-X-box binding protein 1 (XBP1) pathways [27]. This activation led to the upregulation of other UPR genes such as *activating transcription factor 3 (ATF3) and DNA damage-inducible transcript 3 (DDIT3/CHOP)* [27]. The induction of the UPR was also shown to be dependent on the activation of the MEK/ERK signaling pathway, since farnesol treatment augmented the phosphorylation of ERK, and the addition of a specific MEK inhibitor suppressed the induction of the UPR and inhibited apoptosis in response to farnesol [27]. Furthermore, it was shown that farnesol also increased the phosphorylation of other MAPK proteins, p38 and JNK, but the activation of p38 did not seem to play a part in the anti-tumor effects of farnesol [27]. On the other hand, inhibition of JNK did not block farnesol-induced UPR, but it did prevent apoptosis of the cells, suggesting that JNK may act downstream of the farnesol-induced MEK/ERK activation of the UPR to trigger apoptosis [27].

### 2.7. Pancreatic Cancer

Farnesol exerted its anti-tumor effects on pancreatic cancer cell lines, MIA PaCa2 and BxPC-3, by inducing a cell cycle arrest and triggering their apoptosis [28,30]. The cells were arrested in the G0/G1 phase of the cell cycle following treatment with farnesol, which was caused by the upregulation of p21 and p27 expression and the downregulation of cyclin A, cyclin B1, and cyclin-dependent kinase 2 (CDK2) [30]. In MIA PaCa2 cells, cyclin D1 expression was also reduced in response to farnesol [30]. In both cell lines, the increased levels of p21 and p27 resulted in augmentation of the p21/p27-CDK2 complex, which prevented the cells from progressing in the cell cycle [30]. BxPC-3 pancreatic cancer cells treated with farnesol had higher expression of Bak protein and an increase in apoptosis than non-treated cells [28]. In addition, MIA PaCa2 human pancreatic cancer cells and PC-1 hamster pancreatic cancer cells also demonstrated inhibition of cell growth following treatment with farnesol [29].

### 2.8. Cervical Cancer

In a study by Wang and colleagues, it was demonstrated that farnesol decreased the cell viability of cervical cancer HeLa cells in a dose- and time-dependent manner [31]. Farnesol also induced a loss in the mitochondrial membrane potential (MMP) and triggered apoptosis of the HeLa cells through the regulation of the PI3K/Akt pathway [31]. Farnesol downregulated the expression of PI3K and p-Akt proteins in HeLa cells in a dose-dependent manner to exert its anti-tumor effects [31].

### 2.9. Oral Squamous Cell Carcinoma (OSCC)

Farnesol was also able to exert its anti-neoplastic effects on OSCC cell lines, OSCC9 and OSCC25 [32]. Treatment with farnesol inhibited cell growth and induced apoptosis of the OSCC cells. There was an increase in the cleavage and activity of caspase-9 and caspase-3, as well as a decrease in the expression of the anti-apoptotic protein, survivin [32]. Furthermore, farnesol treatment upregulated the expression of several proteins involved in the suppression of carcinogenesis, such as Met-enkephalin, which is involved in the tumor immune function, and Mdm-2, a potent growth suppressor [32]. There was also a downregulation of several oncogenic proteins such as heat shock protein 27 kDa, RAN, and cofilin-1, which can function to inhibit apoptosis, induce genetic instability, cell cycle progression, and promote tumor metastasis [32].

It has previously been established that farnesol can inhibit the synthesis of PC, the precursor of DAG, a lipid second messenger [10]. The addition of exogenous DAG was shown to block the cell growth inhibition induced by farnesol as well as reduce the cleavage of caspase-9 and -3 to their basal levels. Therefore, it is likely that farnesol suppressed the production of DAG in OSCC cells to exert its anti-tumor effects [32].

### 2.10. Meningioma

Farnesol was also able to exert its cytotoxic effects on eleven primary meningioma cell lines of different World Health Organization grades and on the stable malignant meningioma IOMM-Lee cell line. The cell viability of the tumor cell lines was significantly decreased to below 10% after treatment with 1.2 μM farnesol for 24 h [33]. Farnesol also triggered apoptosis of the tumor cells in a dose- and time-dependent manner, where there was an increase and decrease in the expression of active and inactive caspase-3 respectively [33]. Single-stranded DNA was detected in the cells, signifying that apoptosis was taking place since the DNA was being denatured [33]. Additionally, IOMM-Lee cells displayed an augmentation in the level of cleaved PARP1, which is a substrate of caspase-3 and is cleaved during apoptosis [33]. Consequently, farnesol was able to inhibit the growth of meningioma by triggering apoptosis of the tumor cells.

### 2.11. Multiple Myeloma

In multiple myeloma U266 cells, farnesol inhibited the activation of transcription factor signal transducer and activator of transcription 3 (STAT3), a protein involved in tumor initiation as well as progression [34] and also downregulated cell proliferation, and the expression of anti-apoptotic related proteins [34]. Farnesol also reduced the expression of various proteins involved in cell proliferation, angiogenesis, and metastasis [56,57].

### 2.12. T Lymphoblastic Leukemia

The cell growth of lymphoblastic leukemia Molt4-hyg cells was inhibited and apoptosis was triggered when treated with farnesol. 75 μM farnesol increased the population of subG1 phase cells in a time-dependent manner, which was diminished upon the addition of the pan-caspase inhibitor, Z-VAD-fmk, to the treatment, indicating that farnesol dependent cell death may be mediated via caspases [35]. Moreover, it was found that farnesol upregulated the cleavage of caspase-9, caspase-3, and PARP, as well as induced a reduction of the MMP, thus increasing the expression of cytosolic cytochrome c in Molt4-hyg cells [35]. These effects were observed in a lesser extent in Molt-4 cells ectopically expressing Bcl-2, determining that farnesol-induced apoptosis of the T lymphoblastic leukemia cells was mediated through the intrinsic apoptotic pathway [35].

In Molt4-hyg cells, it was found that farnesol augmented the phosphorylation of eIF2α and the expression of several downstream unfolded protein response (UPR) genes, like activating transcription factor 3 (*ATF3*), DNA damage-inducible transcript 3 (*DDIT3* or *CHOP*/*GADD153*), and homocysteine-inducible, ER stress-inducible, ubiquitin-like domain member *2* (*HERPUD2*) [35]. Farnesol caused downregulation of the UPR-related gene, *GRP78*, which can function as an anti-apoptotic protein to rescue the cells from ER stress [35]. In addition, farnesol treatment upregulated the phosphorylation of the MAPK proteins, ERK1/2, p38, and JNK [35]. Other studies have revealed that the UPR may be induced through the activation of the MAPK pathway, but in the case of farnesol-treated Molt4-hyg cells, the specific inhibition of the MAPK pathways did not result in a significant change in the expression of UPR genes compared to the control, signifying that farnesol-induction of the UPR may be independent of the MAPK pathway [27,35]. Thus, farnesol can exert its anti-tumor effects on Molt4-hyg cells partly through the induction of ER stress and activation of the UPR to trigger their apoptosis.

## 3. In Vivo Anti-Inflammatory and Anti-Cancer Effects of Farnesol

### 3.1. Anti-Inflammatory

Farnesol has been demonstrated to inhibit Ras protein and NF-κB activation and induce apoptosis in several in vivo studies (Table 3) [14,17,18,19,20,21]. Farnesol significantly downregulated the Ras-Raf-ERK1/2 signaling pathway, which plays a key role in promoting cell survival, though this pathway can also play a role in growth inhibition and cell death [18,43,59]. Interestingly, a lower dose of farnesol was found to significantly inhibit the Ras-Raf-ERK1/2 signaling pathway, leading to the suppression of inflammatory gene expression, whereas a higher dose led to activation of the Ras-Raf-ERK1/2 signaling pathway in a skin tumorigenesis mouse model [18]. In addition, farnesol increased the Bax/Bcl-2 ratio, which led to induction of apoptosis in a skin tumorigenesis mouse model [18]. In asthmatic mice and gliosis-induced Swiss albino mice, farnesol supplementation decreased IL-6, TNF-α, and inducible nitric oxide synthase (iNOS), demonstrating that farnesol has an anti-inflammatory potential [14,21]. Other studies demonstrated that farnesol could provide lung protection by lowering the levels of lactate dehydrogenase in cigarette smoke extract-treated rats and B(a)P-induced pulmonary injury rats [17,20]. Moreover, other studies have reported decreased caspase-3 activity in 1,2-dimethylhydrazine induced colonic damage rats following treatment with farnesol [19].

### 3.2. Anti-Cancer

#### 3.2.1. Pancreatic Tumor

Farnesol can also abrogate tumor growth and/or reduce oxidative damage in various animal models in vivo [22,29,34]. In PC-1 pancreatic tumor xenograft hamster models, farnesol was added as a dietary supplement of 20 g/kg for 20 days [29]. Control group hamsters were fed a control diet for 20 days, and then compared with the farnesol fed group [29]. Farnesol attenuated tumor growth and the average tumor diameter of the farnesol treated cohort was significantly lower than that of the control on day 25 [29].

#### 3.2.2. Prostate Tumor

Five-week-old male nude mice were injected subcutaneously with DU145 prostate cancer cells into their right flanks and divided randomly into two groups [22]. The farnesol treated group had 50 mg/kg oral treatment every day for 5 weeks [22]. After 29 days, the farnesol treated group demonstrated reduction in tumor growth, which was exemplified by smaller size and lower weight compared to the tumors in the control group [22]

#### 3.2.3. Multiple Myeloma

Six-week-old female nude mice were injected with U266 multiple myeloma cells into the right flank and randomly divided into four groups [34]. The control group (group I) was treated with PBS 200 µL three times for 3 weeks, group II was treated with 60 mg/kg of farnesol three times for 20 days, group III was treated with the anti-cancer drug bortezomib 0.25 mg/kg once a week, and group IV was treated with farnesol (60 mg/kg) and bortezomib (0.25 mg/kg) in combination [34]. Each group was treated for 20 days by intraperitoneal injection, and tumor volume was measured for 5-days interval for 25-days [34]. Group II, III, and IV had significantly decreased tumor volume. Following excision, Immunohistochemistry showed that farnesol treatment reduced the levels of p-STAT3 and Ki-67, while western blot analysis indicated that it also suppressed STAT3 activation in order to induce apoptosis [34].

## 4. Conclusions and Future Perspectives

Overall, several studies have demonstrated the potential pro/anti-inflammatory and anti-cancer effects of farnesol in various diseases and cancers. Although farnesol displayed a more pro-inflammatory effect under in vitro settings, in vivo findings showed that it is likely to act in an anti-inflammatory fashion in various chronic inflammation-induced diseases such as asthma. This could be attributed to inflammation being a process that is dependent on the extracellular milieu, where various types of immune cells also contribute to a pro- or anti-inflammatory environment. Therefore, it is necessary to further investigate the in-depth mechanism of action of farnesol in modulating inflammation.

Additionally, studies have also revealed farnesol to be efficacious as a potential anti-cancer agent as it can exert cytotoxic effects against various neoplastic cell lines and it can significantly inhibit tumor growth in vivo. Farnesol can also regulate various pathways to exert its anti-cancer effects in tumor cells, which include the PC biosynthesis, MEK/ERK, UPR, JAK/STAT3, and PI3K/Akt signaling cascades. Since in most cancers multiple oncogenic pathways would be deregulated to contribute to the progression of the disease, farnesol could be beneficial as it can target different signal transduction pathways, making it a promising anti-cancer therapeutic. However, more research needs to be carried out to determine the safety and efficacy of farnesol for cancer treatment, which includes testing it in clinical trials, as its effectiveness, has not yet been exactly determined in cancer patients.

## Figures and Tables

**Table 1:** Diverse plant sources of farnesol.

Plants	Scientific Name	Usages	Ref.
Citronella	*Cymbopogon nardus*	Source of soap, candles and incense, perfume, cosmetics, and flavoring	[5]
Lemon Grass	*Cymbopogon citratus*	Sterilization Relieves sweating, fever, abdominal pain, and controls skin secretions	[5]
Tuberose	*Polianthes tuberosa* L.	Source of perfume	[5,6]
Cyclamen	*Cyclamen persicum*		[7]
Rose	*Rosa hybrida*	Source of perfume	[8]
Neroli	*Citrus aurantium* subsp. *amara*	Source of perfume	[9]
Balsam		Source of medicinal products, fragrances for varnishes and lacquers, air freshener perfume, and natural remedy for skin rashes	[5]
Musk	*Abelmoschus moschatus*	Source of perfume	[6]

**Table 2:** Molecular targets of farnesol in diverse tumor cell lines.

Origin	Cell Lines	Molecular Targets	Mechanism of Actions	ED_50_ (μM)	Ref.
Prostate	DU145	PI3K/Akt, MAPK ↓	apoptosis ↑	30 and 60	[22]
	LNCaP	Ras	apoptosis ↑, cell proliferation ↓	75	[23]
Breast	MCF-7	Farnesoid X receptor	anti-estrogens ↑, cell growth ↓	10 and 20	[24]
Lung	A549	CPT activity ↓ PC biosynthesis ↓	cell growth ↓ apoptosis ↑, G0/G1 phase arrest ↑	4.5 or 80	[25,26]
	H460	NF-κB ↑ MEK1/2, ERK1/2, UPR ↑	Immune response ↑ cell growth ↓, apoptosis ↑	4.5 or 250	[16,25,27]
Pancreatic	BxPC-3	Bak ↑; p21, p27 ↑, Cyclin A, cyclin B1 ↓ CDK2 ↓	apoptosis ↑ G0/G1 phase arrest ↑	30, 60 and 90	[28,30]
	PC-1	Bak ↑	apoptosis ↑	30, 60 and 90	[29]
	MIA PaCa2	Bak ↑; p21, p27 ↑, Cyclin A, cyclin B1 ↓, Cyclin D1, CDK2 ↓	apoptosis ↑ G0/G1 phase arrest ↑	25, 30, 60 and 90	[29,30]
Cervical	HeLa	PI3K, p-Akt ↓	apoptosis ↑	33.5, 23.8 and 17.6	[31]
Oral squamous cell	OSCC9	Cleaved caspase-9 & -3 ↑	cell growth ↓, apoptosis ↑	30 and 60	[32]
	OSCC25	Cleaved caspase-9 & -3 ↑	cell growth ↓, apoptosis ↑	30 and 60	[32]
Meningioma	Primary meningioma cells	Cleaved caspase-3 ↑	apoptosis ↑	0.4 and 8	[33]
	IOMM-Lee	Cleaved caspase-3 ↑ Cleaved PARP ↑	apoptosis ↑	0.4 and 8	[33]
Multiple myeloma	U266	STAT3, JAK ↓	apoptosis ↑, cell proliferation ↓	100	[34]
	MM.1S	STAT3, JAK ↓	apoptosis ↑, cell proliferation ↓	100	[34]
T lymphoblastic leukemia	Molt4-hyg	*p*-eI2Fα, ATF3, ATF4, CHOP, CHAC1 ↑ (UPR ↑) GRP78 ↓	ER stress ↑ apoptosis ↑	75	[35]
Macrophage	RAW264.7	TNF-α, IL-6 ↑	Immune response ↑	5	[15]
	Primary murine peritoneal macrophage	IL-12 ↓	Inflammation ↓	100	[45]

**Table 3:** In vivo studies with farnesol in diverse diseases.

Disease	Animal	Molecular Targets	Mechanism(s) of Action	Dose and Day of Administration	Ref.
Allergic asthmatic	Mouse	TNF-α ↓ IL-6, IL-10 ↓	Inflammation ↓	5, 25 and 100 mg/kg/day for 5 weeks	[14]
Lung chemoprevention	Rat		Lung prevention ↑	50 and 100 mg/kg/day for 7 days	[17]
Skin tumorigenesis	Mouse	Ras, Raf, ERK1/2 ↓ Bax/Bcl-2 ↑	Inflammation ↓ Apoptosis ↑	25, 50, and 100 mg/kg for 3 days	[18]
Colon carcinogenesis	Rat		Apoptosis ↑	50 and 100 mg/kg for 7 days	[19]
Lung inflammation, Edema	Rat	B(a)P ↓	Lung protection ↑	100 and 200 mg/kg/day for 14 days	[20]
Gliosis	Mouse	iNOS ↓ TNF-α ↓	Inflammation ↓	100 mg/kg for 4 weeks	[21]
Pancreatic cancer	Hamster		Tumor growth ↓	20 g/kg diet for 20 days	[29]
Prostate cancer	Mouse	PI3K/Akt ↓	Apoptosis ↑	50 mg/kg daily for 5 weeks	[22]
Multiple myeloma	Mouse	p-STAT3 ↓	Cell proliferation ↓ Apoptosis ↑	60 mg/kg 3 times/week for 3 weeks	[34]
Oxidative damage to prostate gland	Rat	Glutathione ↓, Antioxidant, enzymes ↑	Xanthine oxidase activity ↓, Lipid hydroperoxide ↓	50 or 100 mg/kg for 7 days	[60]

## Abbreviations

Akt	Serine/threonine kinase
ATF	Activating transcription factor
CDK2	Cyclin dependent kinase 2
CHAC1	Cation transport regulator-like protein 1
COX-2	Cyclooxygenase-2
CPT	Choline phosphate transferase
DAG	Diacylglycerol
DDIT3	DNA damage-inducible transcript 3
ER	Endoplasmic reticulum
IL	Interleukin
iNOS	Inducible nitric oxide synthase
IRE1	Inositol requiring protein 1
MAPK	Mitogen-activated protein kinase
MMP	Mitochondrial membrane potential
NF-κB	Nuclear factor kappa-light-chain-enhancer of activated B cells
OSCC	Oral squamous cell carcinoma
PARP	Poly (ADP-ribose) polymerase
PC	Phosphatidylcholine
PERK	PKR-like ER kinase
PI3K	Phosphatidylinositol-3-kinase
PPAR	Peroxisome proliferator activated receptor
STAT3	Signal transducer and activator of transcription 3
THR	Thyroid hormone receptor
TNFα	Tumor necrosis factor alpha
UPR	Unfolded protein response
XBP1	X-box binding protein 1
↓	Downregulation
↑	Upregulation
ED_50_	Effective dose 50
